# Quantifying Light-Absorbing Aerosol Snow Darkening Using Cryogenic Snow Generation and Integrating Sphere Spectrophotometry

**DOI:** 10.3390/s26206650

**Published:** 2026-10-21

**Authors:** Ganesh S. Chelluboyina, Longrui Zhang, Shu-Wen You, Rajan K. Chakrabarty

**Affiliations:** Department of Energy, Environmental, and Chemical Engineering, Washington University in St. Louis, St. Louis, MO 63130, USA; longrui@wustl.edu (L.Z.); shu-wen@wustl.edu (S.-W.Y.)

**Keywords:** snow albedo, dark brown carbon, light-absorbing particles, SNICAR model, cryogenic synthesis, integrating sphere, aerosol-cryosphere interactions

## Abstract

The deposition of light-absorbing aerosols like dark brown carbon (d-BrC) accelerates cryospheric melt, yet accurately modeling this radiative forcing is hindered by a lack of empirical data. To address this gap, we developed a novel, low-footprint laboratory snow synthesis and deposition apparatus. This system couples the cryogenic generation of synthetic snow with controlled aerosol dry deposition, allowing spectral albedo reductions to be quantified via an integrating sphere spectrophotometer. The setup was rigorously validated using Cabojet, a highly absorbing black carbon (BC) proxy, achieving high-fidelity optical closure with the Snow, Ice, and Aerosol Radiative (SNICAR) model (root-mean-square error < 0.022) and establishing a 165 parts per billion (ppb) detection limit of BC in snow. Applying this validated methodology to nebulized d-BrC tarballs revealed that the dry deposition of ∼1000 ppb d-BrC drives a visible broadband albedo decrease of 0.06. This apparatus offers a highly controlled, empirical platform to ground-truth theoretical radiative forcing calculations for diverse, real-world cryospheric contaminants. Further, by introducing a modular and rapid laboratory setup, these experiments overcome the limitations of outdoor field studies and resource-intensive cold rooms.

## 1. Introduction

The deposition of light-absorbing particles (LAPs) onto the cryosphere triggers a powerful snow-darkening effect by dominating visible broadband albedo reductions [1], exerting a baseline global annual mean surface radiative forcing of +0.054 W m^−2^ (range 0.007–0.13 W m^−2^) [2,3]. Modern deposition profiles demonstrate an increasingly complex LAP burden on global snowpacks characterized by highly diverse aerosol mixtures. This includes black carbon concentrations in mid-latitude regions that exceed polar baselines by up to two orders of magnitude, driving nearly 20% of the albedo reduction across the Tibetan Plateau [4]. Biomass and fossil fuel burning produce plumes of smoke that can travel thousands of kilometers before depositing light-absorbing carbon on snow and ice [5]. Furthermore, this burden is compounded by mineral dust, which dominates high-altitude snow darkening over High Mountain Asia [6] as massive storms from the Taklamakan Desert persistently darken surrounding mountain snowpacks across multiple seasons [7]. This complexity is exacerbated by the emergent accumulation of microplastics across the Arctic, Antarctic, and alpine cryosphere [8]. Despite these substantial regional impacts, the Intergovernmental Panel on Climate Change (IPCC) [9] notes that most CMIP6 climate models do not explicitly represent the darkening of snow by the deposition of this complex aerosol mixture, leading to a systemic underestimation of global radiative forcing.

Beyond this broad mixture of aerosols, the specific parameterization of carbonaceous particles constitutes a major source of uncertainty, primarily due to the intricate brown-black continuum of light-absorbing combustion aerosols [10]. Along this continuum, the imaginary part of the refractive index (*k*) varies by orders of magnitude, progressing from weakly absorbing brown carbon (BrC; k=0.004 at 550 nm) to strongly absorbing dark brown carbon (d-BrC; k=0.25 at 550 nm) depending on the combustion conditions. Recent aircraft measurements and global assessments have revealed the ubiquity of d-BrC in wildfire plumes, demonstrating that, although its mass absorption cross-section (0.5 to 1.5 m^2^ g^−1^ at 500 nm) is lower than that of black carbon (BC), its overall shortwave absorption frequently rivals or exceeds that of BC, challenging the traditional paradigm of BC absorption dominance [11,12]. Consequently, the deposition of d-BrC has emerged as a potent, yet critically under-evaluated, radiative forcing agent in global snowpacks. A recent modeling study [13] established that d-BrC deposition alone can increase annual mean snow radiative forcing by 0.6 to 17.9 W m^−2^, a 1.6- to 2.1-fold enhancement over scenarios considering only black carbon deposition. Given that current models struggle to capture these dynamics, He [14], asserts that “more measurements of BrC optical properties and concentrations in snow are needed” to effectively constrain model estimates. This explicit gap necessitates the development of controlled empirical methodologies capable of precise laboratory deposition.

While robust methodologies have been developed for laboratory sea-ice generation [15,16], the controlled synthesis of snow presents significantly greater logistical challenges. Conventional approaches to actual snow generation typically necessitate resource-intensive, walk-in cold rooms to house massive experimental chambers, such as those requiring 24-h prolonged crystal growth times to produce merely a few kilograms of snow [17,18]. Conversely, techniques circumventing indoor facilities rely heavily on naturally freezing outdoor environments, tethering experimental feasibility to unstable ambient temperatures and weather conditions [19]. Consequently, attempts to study aerosol deposition on snow often suffer from a lack of strict microphysical control; for instance, recent efforts to quantify the effects of BrC deposition were constrained by their reliance on outdoor natural snowpacks, where the lack of environmental containment limits precise optical baselining [20].

To overcome these limitations, the field desperately requires a simple, low-footprint method to simultaneously generate snow and conduct precise, controlled aerosol deposition within a standard, ambient-temperature laboratory. The direct precursor to our apparatus was introduced by Hadley and Kirchstetter [21], who utilized a liquid nitrogen-based methodology to synthesize snow doped with BC. By spraying an aqueous aerosol suspension into a 1.3 m tall insulated chamber cooled to −100 °C, their technique inherently trapped the aerosols within the ice matrix during the phase change. This internal freezing mechanism fundamentally differs, both physically and optically, from true atmospherically relevant dry deposition, wherein aerosols are deposited onto the surfaces of existing snow grains. This is especially relevant as dry deposition flux has been found to be more than five times greater than wet deposition flux at Changbai Mountain, northeastern China [22]. Building upon this foundation, our custom-built, low-footprint apparatus overcomes these spatial and logistical friction points. By facilitating true aerosol dry deposition onto laboratory-generated snow, the system enables highly controlled optical investigations into the radiative forcing of light-absorbing aerosols without the need for expansive cold rooms or outdoor environments.

## 2. Materials and Methods

### 2.1. Laboratory Snow Generation and Aerosol Deposition

**Figure 1:**
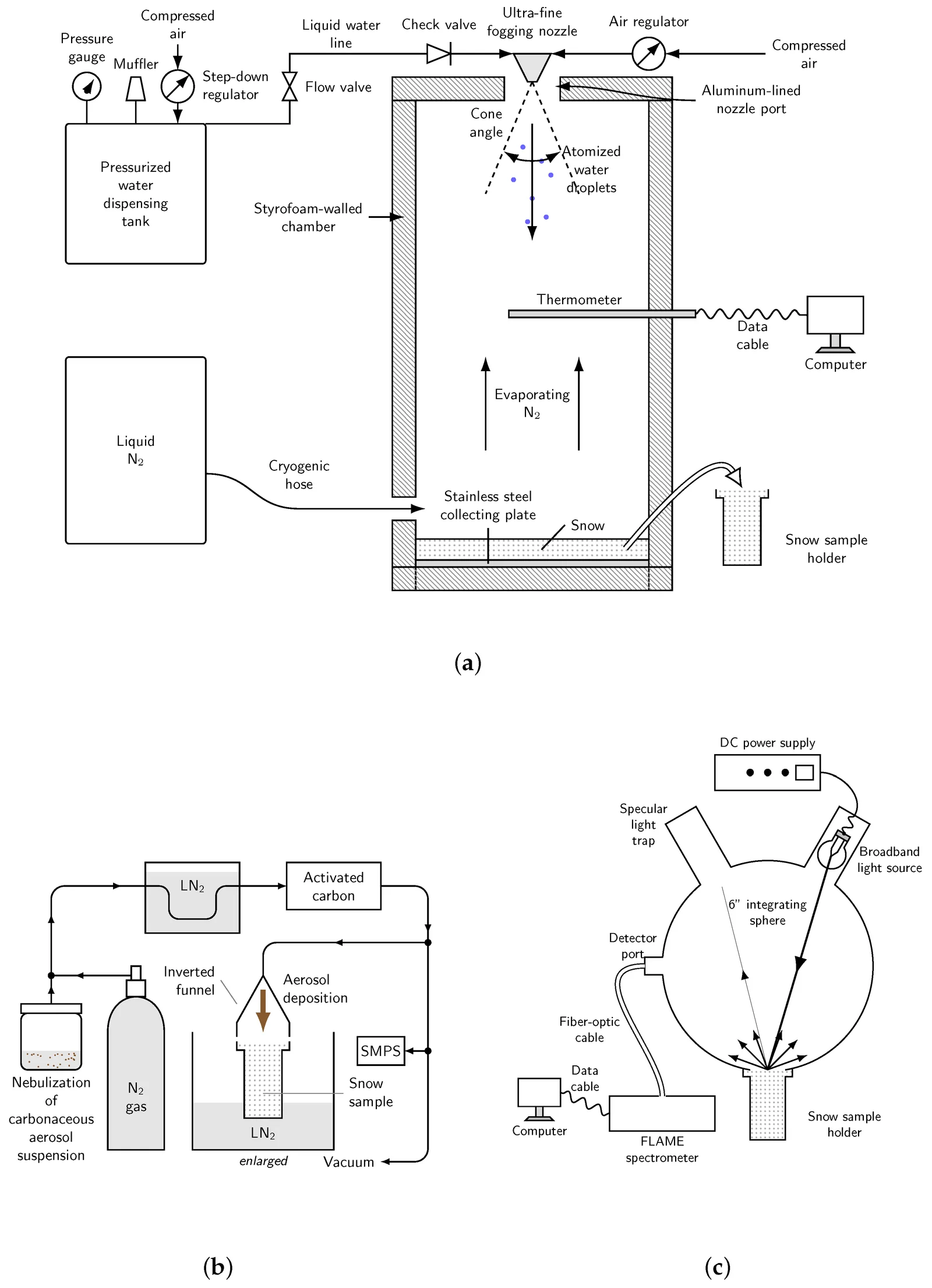
Experimental setup for evaluating the radiative forcing of light-absorbing aerosols on snow. (**a**) Schematic overview of the custom laboratory apparatus used for synthesizing snow under cryogenic conditions. Arrows depict the flow of fluids. (**b**) Detailed schematic of the dry deposition setup used to deposit d-BrC tarball aerosols onto the snow surface. Arrows depict aerosol flow. (**c**) Configuration of the integrating sphere reflectance spectrophotometer in the 8°/d geometry: the collimated beam strikes the sample at 8° incidence, a light trap removes the specular reflection, and the sphere collects the reflected light over the hemisphere; the reference and the sample are measured in the same port by single-beam substitution. Arrows depict the propagation of light rays.

To precisely study the radiative impacts of light-absorbing aerosols on snowpack in a controlled setting, we developed a custom-built laboratory snow generation and deposition apparatus. The system simulates natural snow accumulation by flash-freezing individually atomized water droplets during gravitational settling. The primary component is a 4-foot-tall, thermally insulated styrofoam snow generation chamber. Liquid nitrogen is introduced into the bottom of the chamber to establish cryogenic ambient temperatures through evaporation, which are tunable down to −120 °C. Water is injected into the top of the chamber from a pressurized liquid dispensing tank through an ultra-fine fogging nozzle (60–200 μm droplet size, ITW Vortec, Cincinnati, OH, USA). The tank pressure is regulated by a step-down pressure regulator, allowing the operator to adjust the liquid feed pressure (typically maintained around 10 psi). To further control the atomized droplet size, a flow control valve is used between the tank and the nozzle. Inside the air-powered nozzle, the pressurized water flow is sheared apart into fine droplets by compressed air (40 psi) at a maximum spray angle of 21°. The compressed air flow through the nozzle also serves to circulate the air within the chamber and increase the liquid nitrogen evaporation rate by preventing pooling at the bottom of the chamber. As the droplets descend through the cryogenic environment, they rapidly freeze into near-spherical ice grains that accumulate at the base of the chamber. A schematic overview of the entire snow generation apparatus is presented in Figure 1a.

Following snow synthesis, snow is collected into multiple sample holders. A flat-surfaced putty knife is used to compact the snow in the sample holder and spread the surface evenly. Aerosols are deposited onto the snow surface in a controlled manner. For this study, we focused on Cabojet 200 (Cabot Corporation, Boston, MA, USA) and d-BrC aerosols. Cabojet 200 is a BC-like material that has been used for BC analysis in ice and snow [23,24] and is deposited on snow samples for system validation and limit of detection calculations. Cabojet 200 suspensions in water of varying dilution ratios are aerosolized using a Collison nebulizer. D-BrC is deposited on snow samples as a case study to demonstrate an application of the system to a less well-known particle. D-BrC aerosol was generated via the piezoelectric nebulization of liquid tar suspension followed by a thermal heat shock in a tube furnace [25]. The aerosol stream is subsequently cooled to prevent localized melting of the snowpack upon impaction. Dry deposition of the d-BrC tarballs onto the snow surface is achieved using an inverted funnel enclosure that is designed to promote macroscopically uniform deposition across the snow surface (Figure 1b). A Scanning Mobility Particle Sizer (SMPS) was inserted in parallel to measure particle size distribution. D-BrC aerosol was nebulized at a flow rate of 0.2 L min^−1^ and diluted by a factor of 20 using nitrogen gas. The total deposition time (ranging from 2 to 4 min) is tuned to vary the surface mass loading of the light-absorbing particles across different samples. During deposition, the snow sample holder is held in a bath of evaporating liquid nitrogen to ensure the snow remains frozen, and a slight air gap is maintained between the inverted funnel and sample holder to allow residual air to flow out.

### 2.2. Integrating Sphere Reflectance Spectrophotometer

We utilized a spectrophotometric technique based on an integrating sphere to quantify the spectral albedo reduction of the contaminated snow. The optical system centers on a 6-inch diameter integrating sphere (RT-060-SF, Labsphere Inc., North Sutton, NH, USA) lined with Spectraflect, a highly reflective Lambertian coating (Figure 1c). The sphere is illuminated by a collimated halogen light source capable of a maximum 10 W output. The source is driven by a programmable DC power supply controller, which allows for precise regulation of the output current and target set points.

Snow samples are collected in custom-designed 3D-printed cylindrical holders with a depth of approximately 5 cm, which exceeds the ultraviolet and visible light penetration depth for snow, thereby ensuring semi-infinite optical thickness. The sample holder is mounted flush against the sample reflectance port of the integrating sphere, which faces downward (Figure 1c). The sphere has five 1-inch (25.4 mm) ports around its equator and a 0.5-inch (12.7 mm) detector port at its pole [26]. The collimated beam enters through the sample beam port and strikes the snow surface at an incidence angle of 8° from the normal of the sample port [26]. The specular exclusion port lies at the mirror image of the incident beam about the sample normal and holds a light trap, which absorbs the specularly reflected beam and returns no light to the sphere. The two unused 1-inch ports are closed with port plugs. The optical fiber of the spectrometer is coupled to the detector port, which is baffled from the sample port; the fiber therefore does not view the directly illuminated sample and receives only light that has been reflected at least once by the sphere wall. The detected signal is thus proportional to the flux that the sample reflects into the hemisphere above it, independent of how that flux is distributed in angle [27]. This configuration is the 8°/d (directional-hemispherical) geometry with the specular component excluded. Spectral reflectance is measured over the 350–1000 nm wavelength range using a high-resolution spectrometer (Flame-S-VIS-NIR, Ocean Optics Inc., Orlando, FL, USA). Measurements were made under the single-beam measurement condition with the reference and sample physically swapped at the same port. Spectra were acquired with the OceanView program (Ocean Optics Inc.) in its reflectance mode, which computes the ratio $\frac{S_{\mathrm{snow}}-S_{\mathrm{dark}}}{S_{\mathrm{std}}-S_{\mathrm{dark}}}$ used in Equation (1) below from stored dark and reference spectra; each spectrum is the average of three scans.

Snow reflects light mainly by multiple scattering among many randomly oriented ice grains, so the reflected light is spread over the hemisphere and contains no concentrated mirror-direction beam. For a diffusely reflecting surface at one port of the sphere, the fraction of its reflected flux that reaches another port equals that port’s area divided by the sphere’s surface area [28]; this fraction is 0.7% for a 1-inch port on the 6-inch sphere. The light trap and the entrance port therefore each remove about 0.7% of the flux reflected by the snow and by the reference standard alike, and this common loss cancels in their ratio. If the snow radiance toward either port were 20% higher than that of a Lambertian reflector of equal reflectance, the uncancelled loss would be about 0.14% of the reflected flux (about 0.001 in reflectance), well below the variability of the pristine-snow measurements (Section 3.2). Excluding the specular component therefore does not change the measured reflectance of snow appreciably.

The measured quantity is the spectral reflectance of the snow, obtained by substitution against the reference standard:

(1) $$R\left(\lambda \right)=\rho_{\mathrm{std}}\left(\lambda \right)\frac{S_{\mathrm{snow}}\left(\lambda \right)-S_{\mathrm{dark}}\left(\lambda \right)}{S_{\mathrm{std}}\left(\lambda \right)-S_{\mathrm{dark}}\left(\lambda \right)},$$

where $S_{\mathrm{snow}}$, $S_{\mathrm{std}}$, and $S_{\mathrm{dark}}$ are the spectrometer signals recorded with the snow sample in the sample port, with the reference standard in the same port, and with the beam blocked, and $\rho_{\mathrm{std}}\left(\lambda \right)$ is the certified reflectance of the standard. The standard is a Spectralon diffuse reflectance standard (SRS-99-010, Labsphere Inc.), whose 8°/hemispherical reflectance was calibrated by the manufacturer, traceably to NIST, from 250 to 2500 nm at 50 nm intervals (calibration report of 2 July 2024). Between 400 and 800 nm, the certified values lie between 0.9890 and 0.9903 with an expanded uncertainty of 0.005 (coverage factor 2), and we interpolate them linearly to the spectrometer wavelengths. Apart from the substitution error treated below, R(λ) is the directional-hemispherical reflectance of the snow for a collimated beam incident at 8°, which radiative transfer theory calls the plane or black-sky albedo [29].

**Figure 2:**
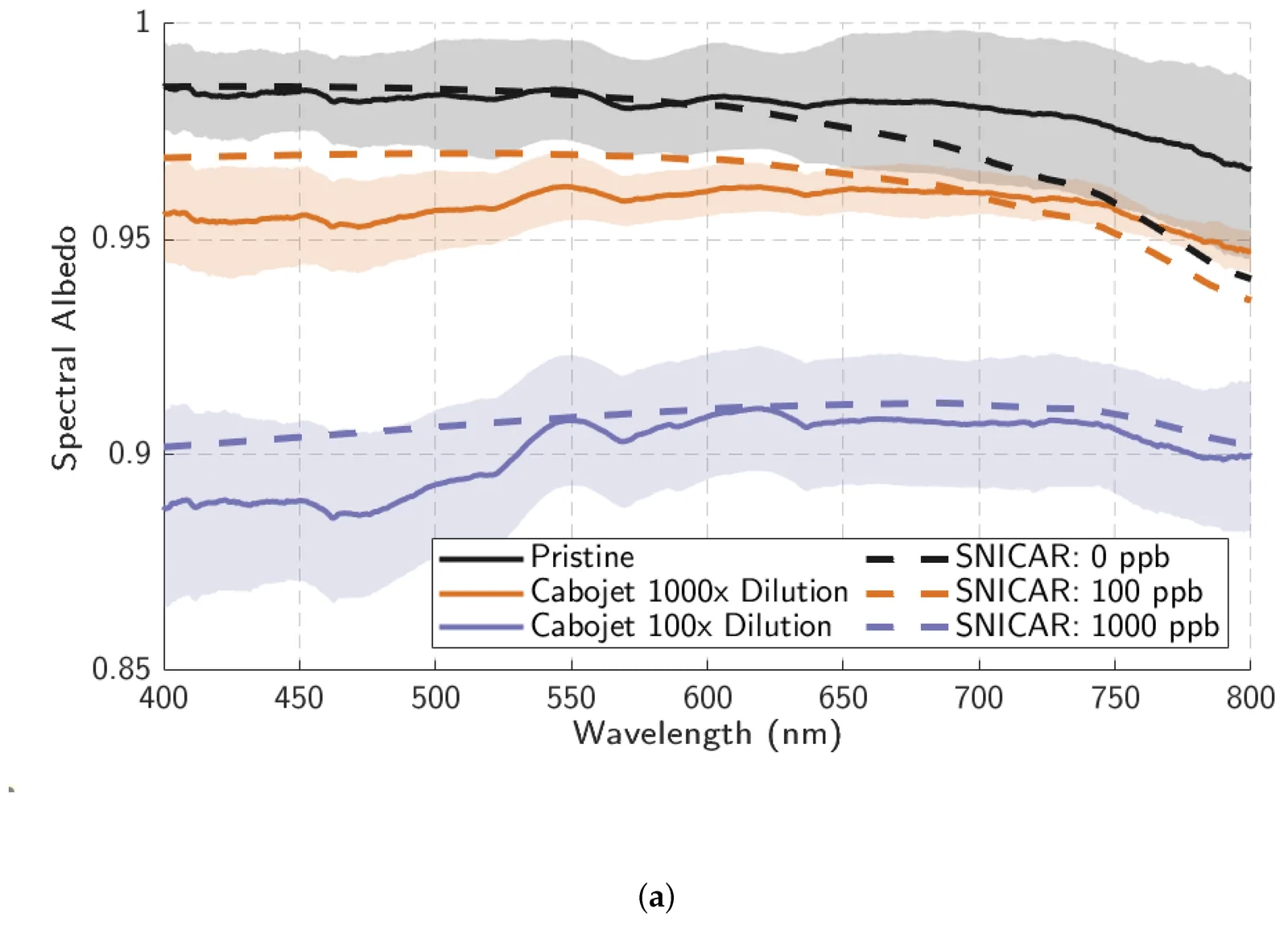

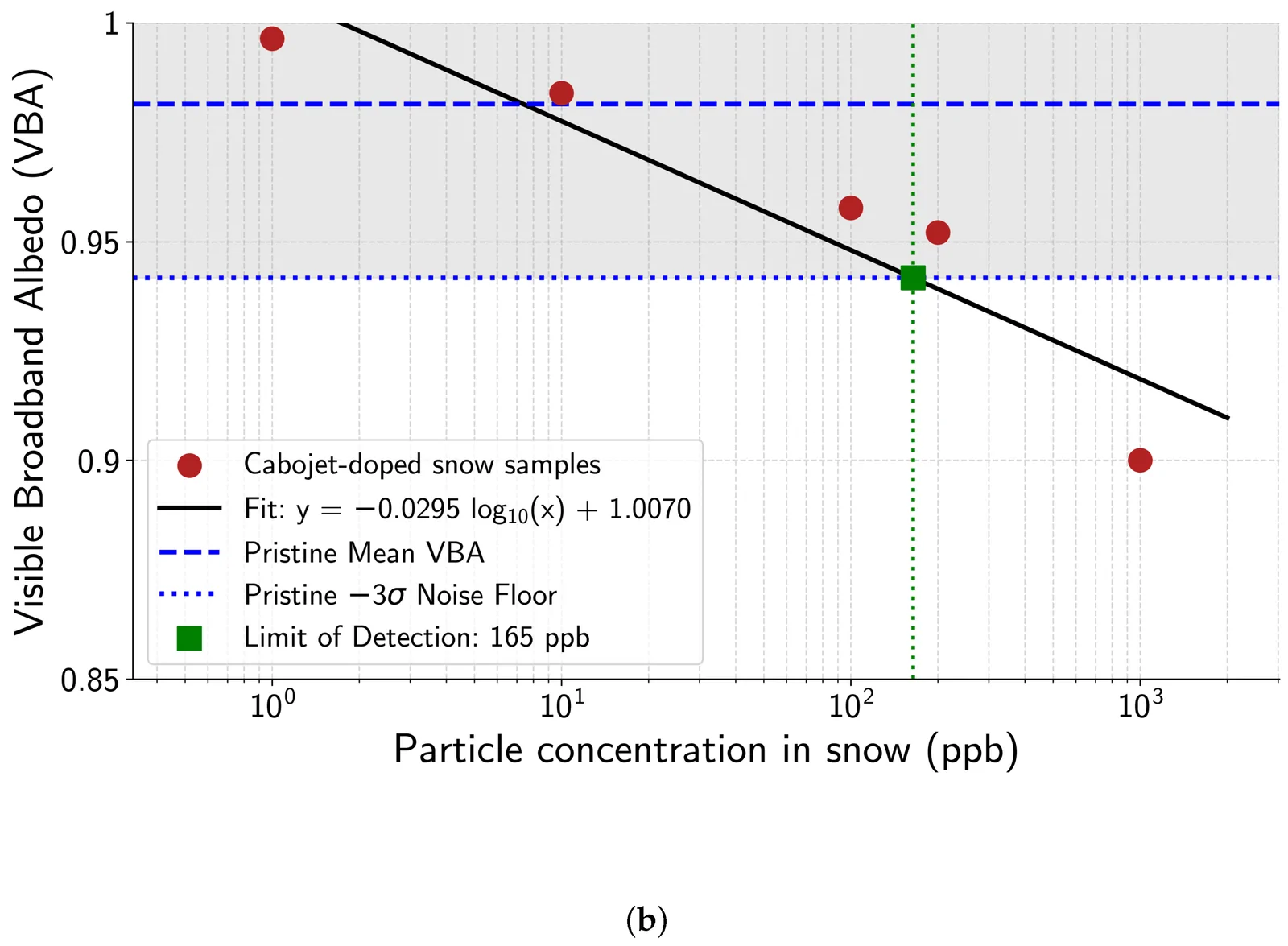
(**a**) Optical closure between experimental Cabojet-doped snow spectra and SNICAR-ADv4 simulations. (**b**) Limit of Detection (LOD) regression analysis for the optical measurement system. The gray shaded region represents the 3σ uncertainty band of the pristine snow visible broadband albedo, and the vertical green dashed line denotes the 165 ppb LOD threshold, determined by the intersection of the regression fit and the noise floor.

**Figure 3:**
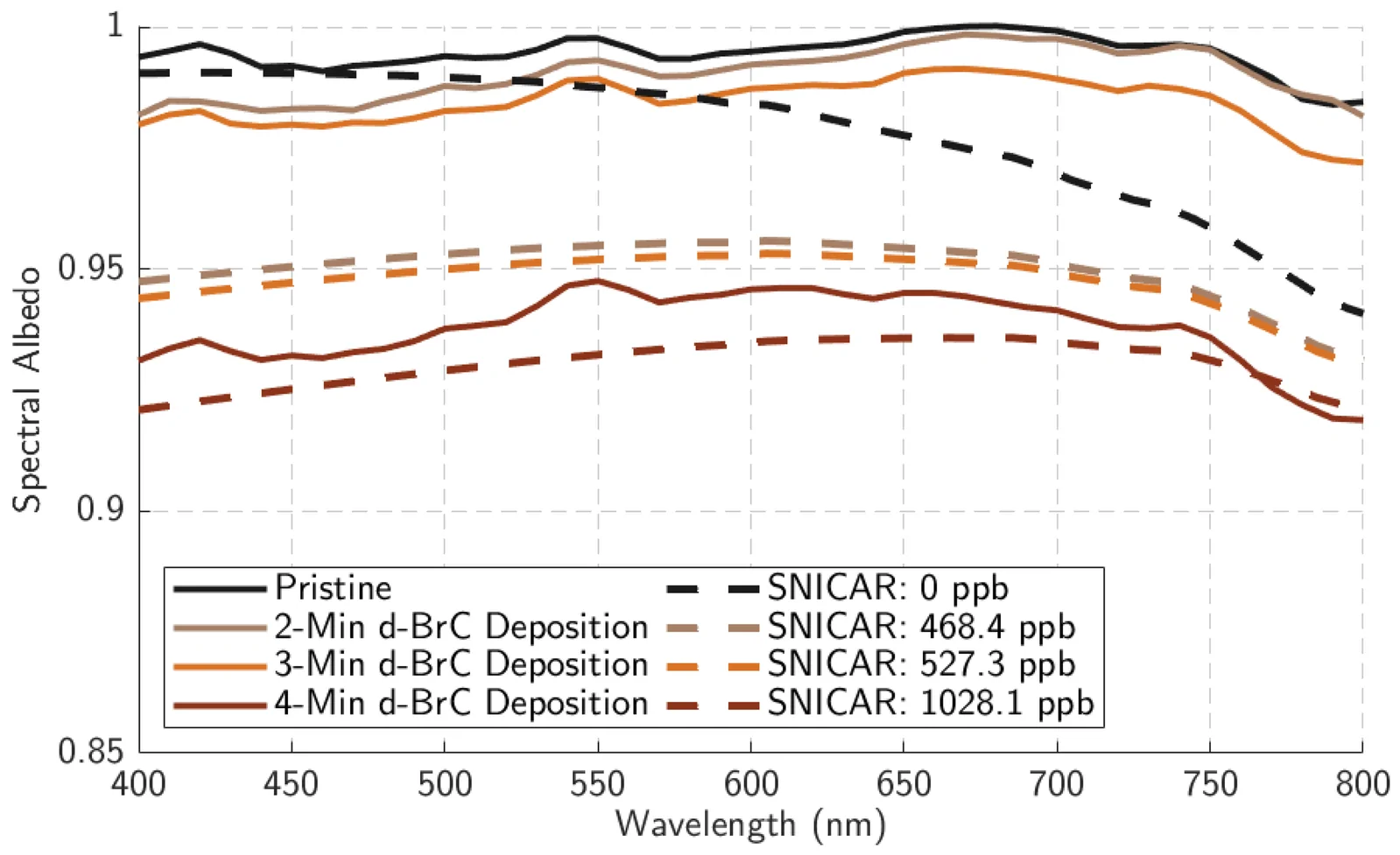
Divergence between experimental d-BrC-doped snow spectra and SNICAR-ADv4 simulations using SMPS-derived mass loadings.

SNICAR computes the same quantity when it is run with direct-beam illumination: the albedo of a plane-parallel snowpack under a collimated beam at solar zenith angle $\theta_{0}$ is the upward hemispheric flux divided by the incident flux. The SNICAR spectra in Figure 2 and Figure 3 are therefore direct-beam albedos computed for $\theta_{0}=8{}^{\circ}$ ($\cos\theta_{0}=0.99$). The diffuse (white-sky) albedo of the same snowpack would not be an equivalent comparison. Under near-normal incidence, photons penetrate deeper into the snowpack before they are scattered back toward the surface and are therefore more likely to be absorbed, so the directional-hemispherical albedo at small $\theta_{0}$ is lower than the diffuse albedo [30]. For weakly absorbing snow, the diffuse albedo $R_{d}$ and the directional-hemispherical albedo *R* at incidence angle $\theta_{0}$ are related by $R_{d}=R^{1/u}$, with $u=\frac{3(1+2\cos\theta_{0})}{7}$ [31]; at $\theta_{0}=8{}^{\circ}$, u=1.28, so a snowpack with R=0.90 has $R_{d}\approx 0.92$.

Because the standard and the sample are measured in the same port, a sample darker than the standard lowers the average reflectance of the sphere wall and therefore the sphere multiplier, so Equation (1) underestimates the reflectance of that sample (single-beam substitution error; [28]). For the port fractions of our sphere and a wall reflectance of 0.97 ± 0.01, the sphere radiance equation [28] gives an error below 0.001 for pristine snow and about −0.010 for the darkest Cabojet sample (R≈0.89). This error is smaller than the standard deviation among the pristine samples (0.013; Section 3.1), and we do not correct for it. Because it causes Equation (1) to overestimate the darkening of the doped samples by up to about 10%, the limit of detection in Section 3.2 is a lower estimate. Throughout this paper, “spectral albedo” refers to R(λ) from Equation (1).

### 2.3. Optical Microscopy and Microphysical Characterization

In parallel with the bulk optical measurements, we performed microphysical characterization of the synthesized snow grains and deposited aerosols. A thin layer of the freshly generated snow was collected on a pre-chilled, improvised aluminum block designed to prevent rapid melting. The snow grains were imaged using a high-magnification digital light microscope. These microscopic observations showed that the laboratory-generated snow consists of near-spherical grains with diameters of approximately 100 μm, consistent with observations of natural fresh snow. The microscopy was also used to examine the distribution and mixing state of the dry-deposited d-BrC tarballs on the snow surface (Section 3.4).

## 3. Results and Discussion

### 3.1. Optical Validation Using Cabojet

To rigorously validate the snow synthesis, aerosol deposition, and optical measurement system, initial experiments were conducted using a standardized, highly absorbing BC proxy (Cabojet). Spectral reflectance was measured for pristine laboratory-synthesized snow and snow doped with sequentially diluted aerosol suspensions (100× dilution all the way up to 100,000× dilution). To isolate the physical optical signature from high-frequency instrumental noise, the raw, uncut reflectance arrays were processed using a Savitzky–Golay filter (window length = 151, polynomial order = 3) and truncated strictly to the 400–800 nm range to avoid near-infrared artifacts from the halogen light source.

The ratio $\frac{S_{\mathrm{snow}}-S_{\mathrm{dark}}}{S_{\mathrm{std}}-S_{\mathrm{dark}}}$ in Equation (1) is close to unity for pristine snow because pristine snow and the Spectralon standard have nearly equal reflectance. After calibration with Equation (1), the mean visible broadband albedo of the pristine samples in Figure 2b is 0.98, and individual pristine spectra exceed unity by at most 0.01 at some wavelengths. These excursions are within the measurement uncertainty, which comprises the variability among the pristine samples (standard deviation of the visible broadband albedo, 0.013; Section 3.2) and the calibration uncertainty of the standard (0.005, coverage factor 2); the spectra are therefore reported without further scaling.

To achieve optical closure, the experimental curves were compared against direct-beam spectral albedos ($\theta_{0}=8{}^{\circ}$; Section 2.2) simulated by the Snow, Ice, and Aerosol Radiative (SNICAR-ADv4) model [32,33] (Figure 2a). Snow samples, which were 5 cm thick, were assumed to consist of two layers: a top layer with aerosol homogeneously mixed and an underlying layer of pristine snow. In the model, the physical structure of the snowpack was locked across all samples to a realistic compacted matrix of snow grains (density 600 kg m^−3^, effective grain radius 50 µm, top layer thickness 10 mm), allowing only the equivalent black carbon (BC) mass concentration to vary.

The homogeneous 10 mm top layer is a simplification. The quantity fixed by the experiment is the deposited mass per unit area: 1 ppb in the 10 mm layer at 600 kg m^−3^ corresponds to 6 μg m^−2^, so the concentrations reported here scale inversely with the assumed mixing depth. For the same deposited mass, the real deposit differs from the simulated layer in three ways. First, dry deposition places the particles at the surface rather than throughout the top 10 mm. Light weakens with depth in snow, so particles near the surface absorb more of it, and a thin surface deposit darkens the snow more than the simulated layer does; the difference grows with loading because the particles themselves absorb light within the layer, but it remains smaller than the effect of the deposited mass itself, because the 10 mm layer is thinner than the depth over which visible light is attenuated in snow [1]. Second, the microscopy (Section 3.4) shows that the deposit is uneven across the surface. Doubling the local concentration less than doubles the local darkening (Figure 2b), so the darkening gained where particles accumulate is smaller than the darkening lost where they are sparse, and an uneven deposit darkens the snow less than the same mass spread evenly. This applies to patches larger than the distance that photons travel sideways in the snow because photons average over smaller patches. Third, the particles aggregate. An absorbing particle absorbs light within a surface shell whose thickness is $\frac{\lambda }{4\pi k}$, about 0.2 μm for d-BrC at 550 nm (k=0.25); the interior of a larger aggregate is therefore shaded and absorbs little, and aggregates absorb less light per unit mass than the dispersed particles assumed in SNICAR. The simulated layer thus underestimates the darkening of a thin, even surface deposit and overestimates that of a patchy or aggregated deposit.

The model successfully reproduced the empirical spectral shapes with high fidelity. The 1000× and 100× dilution curves matched SNICAR outputs for 100 ppb and 1000 ppb of BC, respectively, yielding an average joint root-mean-square error (RMSE) of under 0.022. The distinct visible-spectrum curvature (400–600 nm) indicative of carbonaceous absorption is perfectly captured. Furthermore, the tenfold increase in modeled mass loading (100 ppb to 1000 ppb) directly mirrors the tenfold sequential dilution of the source suspension. This optical closure confirms that the deposition system operates with high predictability and linearity and that the laboratory-generated snowpack responds to radiative forcing in accordance with established physical theory.

### 3.2. Limit of Detection (LOD) and System Boundaries

Having established optical closure, the boundaries of the measurement system were quantified. For atmospheric and cryospheric applications, integrating spectral data into a single broadband albedo metric is necessary to evaluate total shortwave energy balances. Consequently, the individual measured spectral albedos were collapsed into visible broadband albedos (VBA) by weighting the spectra by the incident irradiance fraction for a standard mid-latitude summer solar spectrum.

To calculate the Limit of Detection (LOD) of the optical measurement system, the 3σ uncertainty band (noise floor) of the pristine snow VBA was established. This standard deviation was calculated across three independent pristine snow samples, thereby representing true sample-to-sample variability rather than strictly instrumental repeatability. A calibration curve was then generated by calculating the VBA for five standardized Cabojet-doped samples, corresponding to equivalent mass loadings of 1, 10, 100, 200, and 1000 ppb. A semi-logarithmic ordinary least squares regression ($y=m\log_{10}\left(x\right)+c$) was fitted to these five broadband albedo data points. As shown in Figure 2b, the LOD was determined at the exact intersection of this concentration regression line and the pristine 3σ noise floor threshold. The resulting BC-equivalent LOD is 164.5 ppb. This threshold indicates that the presented methodology is capable of detecting and quantifying the particulate darkening effect in moderately to heavily polluted snowpacks, establishing operational boundaries for future field-mimicking experiments.

### 3.3. Application to d-BrC Deposition

Following system validation, the methodology was applied to a complex, novel aerosol: nebulized liquid tar d-BrC. The d-BrC deposition was conducted in parallel with an SMPS. During the controlled deposition, the aerosol stream was split evenly. A flow rate of 0.2 L min^−1^ was directed to the SMPS, and 0.2 L min^−1^ was directed to the snow sample via the inverted funnel. The SMPS-derived number size distributions were converted to aerosol snow mass mixing ratios assuming an 80% deposition efficiency within the enclosed funnel alongside a d-BrC particle density of 1.4 g cm^−3^. The integrated size distributions were converted into total deposited aerosol mass based on the individual sample deposition durations of 2, 3, or 4 min.

Using these mass loadings, forward SNICAR simulations were performed assuming homogeneous and external mixing of the aerosol in the topmost layer of snow (Table 1). These simulations were executed assuming direct illumination under clear-sky conditions. The aerosol snow mass mixing ratio calculation utilized a uniform localized deposition area derived from the 1 inch inner diameter of the funnel. The snow density of 600 kg m^−3^ aligns with the theoretical random close packing fraction (∼64%) of solid ice spheres and reflects the physical state of the mechanically compacted frozen droplets. Furthermore, it closely matches the 550 kg m^−3^ density reported by Hadley and Kirchstetter [21] for their analogous laboratory snow generation chamber. Based on these parameters, the deposited d-BrC yielded mass mixing ratios of 468.4 ppb, 527.3 ppb, and 1028.1 ppb, respectively. For the heaviest loading of 1028.1 ppb, the experimental visible broadband albedo decreased by 0.06 relative to pristine snow. Because the imaginary refractive index of d-BrC ($k_{550}=0.25$) is substantially lower than that of BC ($k_{550}\approx 0.79$), a higher mass loading of d-BrC is required to exert the same macroscopic snow-darkening effect.

As shown in Figure 3, while the heaviest deposition case (1028.1 ppb) showed reasonable agreement in the short-visible wavelengths, at lower deposition masses close to the limit of detection, modeled albedos diverged from experimental albedos, particularly at longer wavelengths (>650 nm).

This divergence suggests the necessity of the empirical methodology presented in this study. SNICAR and similar radiative transfer models assume ideal, spherical particles with neat external or internal mixing states embedded uniformly within an infinite ice matrix. The divergence of the model from the SMPS-derived d-BrC curves suggests that the morphological, mixing, and refractive properties of d-BrC are more complex than what standard parameterizations accurately predict.

### 3.4. Microphysical Evidence of Aerosol Deposition

**Figure 4:**
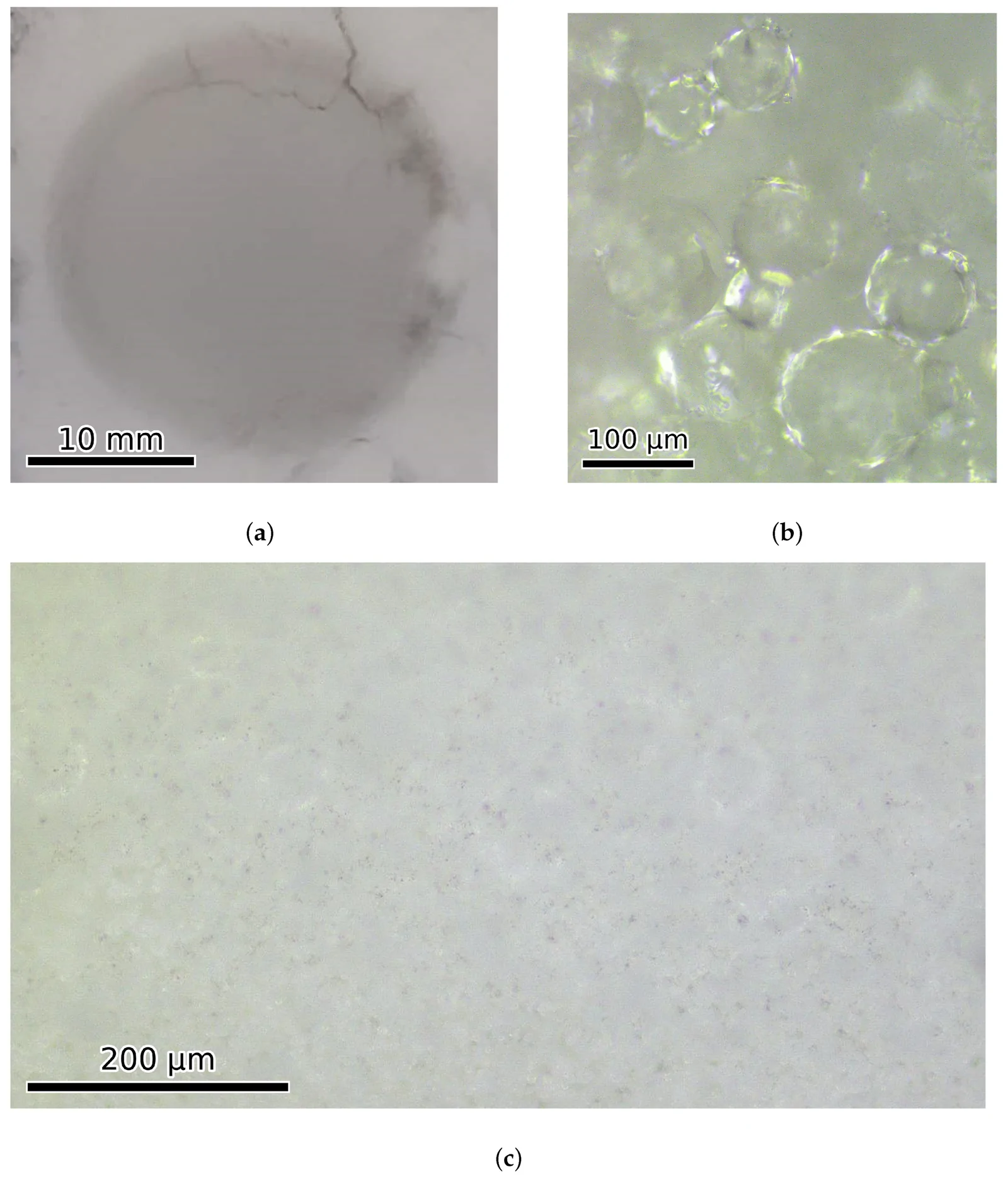

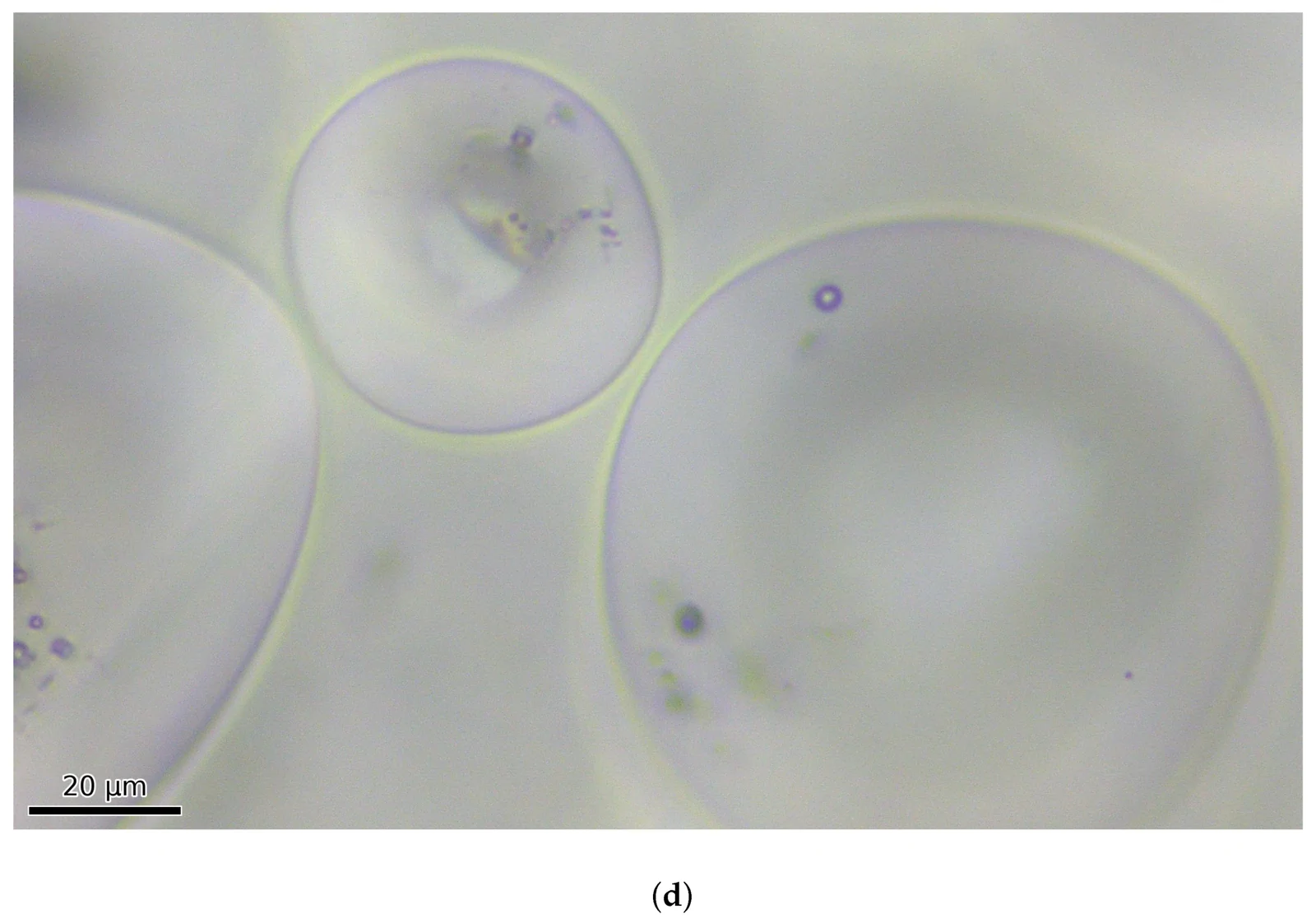
(**a**) Macroscopic image of a circular patch of d-BrC-deposited snow surrounded by pristine snow. (**b**) Optical microscopy showing pristine laboratory snow grains. (**c**) Optical microscopy view showing deposition of d-BrC over the snow crystal matrix in the foreground, with the outlines of snow grains visible in the background. (**d**) Zoomed-in view of snow grains showing bubbles and particles.

Optical microscopy was performed on the snow samples (Figure 4) to observe snow grain shape and size, as well as aerosol deposition. The microscopy aligns with the structural assumptions used in the SNICAR modeling, including that pristine snow grains (Figure 4b) exhibit roughly spherical geometries on the order of 100 μm in diameter, supporting the 50 μm effective radius parameter utilized in the optical closure. Circles fitted to the outlines of the nine grains that are fully visible in the micrograph from which Figure 4b is cropped have diameters of 61–133 μm (mean 89 μm, standard deviation 23 μm), within the 60–200 μm droplet size range of the nozzle (Section 2.1). Their effective radius $r_{\mathrm{eff}}$, the ratio of the third to the second moment of the radius distribution, is 50 μm. For spheres, the specific surface area (SSA), the surface area of ice per unit mass, is $\mathrm{SSA}=\frac{3}{\rho_{ice}r_{\mathrm{eff}}}$, where $\rho_{ice}=917$ kg m^−3^ is the density of ice; the laboratory snow therefore has an SSA of about 65 m^2^ kg^−1^. This value lies within the range observed in natural seasonal snow, from 2 m^2^ kg^−1^ for melt–freeze layers to 156 m^2^ kg^−1^ for fresh dendritic snow [34].

The near-spherical shape follows from how the grains form. Natural snow crystals grow from water vapor while they fall through clouds, and this vapor-deposition growth produces their dendritic and other faceted shapes [35]. In the chamber, the grains instead form by the freezing of liquid droplets, which surface tension holds spherical. A droplet freezes within about a second in the cryogenic air and reaches the base of the chamber within seconds, too soon for vapor deposition to change its shape, so each grain keeps the shape and size of the droplet from which it formed. Hadley and Kirchstetter [21] obtained spherical grains with the same freezing-droplet method and noted that such grains resemble aged natural snow grains, which have been rounded by metamorphism [36], more closely than freshly fallen snow. Spherical grains are also assumed in our SNICAR simulations, so grain shape does not bias the model–measurement comparison in Section 3.1.

However, images of the d-BrC-deposited snow (Figure 4c,d) reveal complex aerosol-snow interactions. The d-BrC particles do not deposit as independent, uniformly distributed spheres; rather, they exhibit clumping, pooling, and localized aggregation along ice crystal boundaries and air bubbles. These non-uniform microphysical changes during the dry deposition of d-BrC may not be adequately captured by standard radiative transfer models. Consequently, these images help underscore the critical need for robust, physical laboratory simulation chambers to empirically measure the true radiative forcing of complex carbonaceous aerosols.

The frozen-droplet snow differs from natural snow in four ways that limit the transfer of our results to natural snowpacks. First, grain shape: for the same SSA, nonspherical grains scatter less light in the forward direction than spheres, which raises the albedo of pure snow and reduces the albedo reduction caused by black carbon [37]. The darkening measured here therefore applies most directly to snow with rounded grains, and transferring it to fresh snow requires accounting for grain shape. Second, grain size: the grain size is set by the droplet size of the nozzle (Section 2.1), so snow with coarse grains, such as aged or melt–freeze snow, cannot be produced directly. Third, bonding: at the cryogenic temperatures of synthesis and deposition, the vapor transport that bonds and reshapes natural snow grains within hours to days [36] is negligible, so the grains remain unbonded; the compacted samples had a bulk density of 600 kg m^−3^. Fourth, air bubbles: the grains contain air bubbles (Figure 4d), which form when air dissolved in the water is expelled during freezing; snow crystals grown from vapor contain none, and scattering by the bubbles is not represented in SNICAR.

## 4. Conclusions

In this study, we presented a novel, low-footprint laboratory apparatus designed for the cryogenic synthesis of laboratory snow from frozen water droplets and the subsequent controlled dry deposition of aerosols. By coupling this generation chamber with an integrating sphere spectrophotometer, we achieved high-fidelity optical closure (average joint RMSE < 0.022) using a standardized carbonaceous proxy, establishing a visible broadband limit of detection of 165 ppb equivalent black carbon. Furthermore, the application of this system to nebulized d-BrC tarballs demonstrated that morphologically complex aerosols form localized aggregates and pools along ice crystal boundaries. These complex microphysical interactions drive nuanced spectral albedo reductions that deviate from the idealized spherical mixing states typically utilized in standard radiative transfer models, underscoring the necessity of empirical methodologies to accurately constrain aerosol radiative forcing.

Beyond static optical validation, the modular, multifunctional, and customizable design of this apparatus provides a robust platform for developing a process-based understanding of aerosol-cryosphere interactions. Future methodological refinements may focus on optimizing deposition flow rates and exposure times to mitigate surface cratering and localized melting during heavy dosing. Additionally, the controlled nature of the chamber enables high-resolution studies on the temporal evolution of snowpack optics, facilitating experiments on snow metamorphism and grain evolution under varying relative humidity conditions and thermal cycling. Further microphysical investigations are also warranted to understand the precise structural and optical implications of liquid N_2_-soaked snow resulting from this specific cryogenic synthesis process.

This laboratory setup offers broad utility for the next generation of cryospheric research. As the recent literature highlights the expanding diversity of light-absorbing impurities, this system is uniquely positioned to investigate the radiative impacts of established as well as emerging contaminants, including microplastics [38], snow algae [39], and other visible-light-absorbing pigments. Snow albedo geoengineering, which has recently attracted attention [40], may also be tested using this system before wider deployment. Furthermore, the lifecycle of these deposited particles can be comprehensively tracked beyond initial deposition through simulated meltwater analysis and subsequent re-aerosolization studies utilizing a Single-Particle Soot Photometer (SP2). By providing an empirical bridge between complex aerosol morphologies and theoretical radiative transfer, this methodology supports a more accurate, holistic understanding of contaminant-driven cryospheric melt.

## Figures and Tables

**Table 1:** SNICAR-ADv4 input parameters used for optical closure simulations.

Parameter	Value
Illumination conditions	Direct beam (clear sky)
Solar zenith angle	8°
Aerosol mixing state	External
Snow grain effective radius	50 μm
Bulk snow density	600 kg m^−3^
Top layer thickness	10 mm
d-BrC imaginary refractive index ($k_{550}$)	0.25

## Data Availability

The data that support the findings of this study are available from the corresponding authors upon reasonable request.
